# Assessment of tear film lipid layer thickness in patients with Meibomian gland dysfunction at different ages

**DOI:** 10.1186/s12886-020-01667-8

**Published:** 2020-10-06

**Authors:** Junhua Li, Jiling Ma, Man Hu, Jianqin Yu, Yune Zhao

**Affiliations:** 1 School of Ophthalmology and Optometry, Eye Hospital, Wenzhou Medical University, 270 West Xueyuan Road, Wenzhou, 325027 Zhejiang China; 2 Key Laboratory of Vision Science, Ministry of Health, Wenzhou, Zhejiang China

**Keywords:** Meibomian gland dysfunction, Lipid layer thickness, Meibomian gland loss, Keratograph, LipiView interferometer

## Abstract

**Background:**

To evaluate the correlations between lipid layer thickness (LLT) and morphology and function of the meibomian glands in patients who were diagnosed as meibomian gland dysfunction (MGD) in different age groups.

**Methods:**

Patients who have diagnosed as obstructive MGD were included in this prospective, cross-sectional study. Patients were divided into three groups: young (ages 20–39 years), middle-aged (ages 40–59 years), and older (aged ≥60 years). All patients completed an Ocular Surface Disease Index (OSDI) questionnaire and were evaluated for LLT, tear meniscus height (TMH), noninvasive tear film break-up time (NI-BUT) measurement, invasive TBUT (ITBUT), corneal fluoresceinstaining (CFS) score, eyelid margin abnormalities, Schirmer I test, and MG function and morphology, by using the Keratograph 5 and LipiView interferometer.

**Results:**

Two hundred and nine patients (209 eyes) were included. The median LLT of all patient was 57 nm (IQR, 36.5 nm), and the LLT values were significantly different among the young group (median, 51 nm; IQR, 23.5 nm), middle-aged group (median, 59.5 nm; IQR 46.5 nm) and older group (median, 62 nm; IQR, 42.5 nm) (*P* = 0.033, Kruskal-Wallis test). In regression analyses controlling for confounder factors sex and MG loss, the LLT was positively correlated with age (β = 5.539, *P* = 0.001). There was a negative correlation between LLT and MG dropout in the all (*r* = − 0.527, *P* < 0.001), young (*r* = − 0.536, *P* < 0.001), middle-aged (*r* = − 0.576, *P* < 0.001), and older (*r* = − 0.501, *P* < 0.001) groups. LLT was positively correlated with the MG expressibility in the all (*r* = 0.202, *P* = 0.003), middle-aged (*r* = 0.280, *P* = 0.044) and older (*r* = 0.452, *p* < 0.001) groups, but it was no statistical significance in the young group (*r* = 0.007, *P* = 0.949).

**Conclusions:**

The thickness of LLT was increased with age and significantly correlated with both MG secretion and morphology in middle-aged and older patients with obstructive MGD. LLT measurement is a useful screening tool for detecting obstructive MGD and age as an influential factor should be accounted for when interpreting the meaning of the LLT value.

**Trial registration:**

NCT02481167; Registered 25 June, 2015.

## Background

Meibomian gland dysfunction (MGD), first described by Korb and Henriquez in the early 1980s, is the most frequent cause of intrinsic evaporative dry eye (EDE), as well as being associated with aqueous-deficient dry eye (ADDE) [1]. MGD is recognized as “a chronic, diffuse abnormality of the meibomian glands, commonly characterized by terminal duct obstruction and/or qualitative/quantitative changes in glandular secretion, and it may result in alteration of the tear film, symptoms of eye irritation, clinically apparent inflammation, and ocular surface disease” [2]. The prevalence of MGD ranges from 3.5% to almost 70% in the worldwide and it is higher in Asians than in Caucasians [3]. Therefore, MGD has been considered as a new public health problem.

In clinical practice, assessing the functional and morphological changes of meibomian gland (MG) is important for diagnosis of MGD. However, although various testing methods are available, most of these techniques are invasive, requiring direct contact with the ocular surface, subjective, and lack of uniform standard [4]. Meibomian lipids are secreted from the MGs and distributed by blinking to form a superficial lipid layer that stabilizes the tear film and prevents tear evaporation [5]. Lipid deficiency due to MGD results in a thinning of the lipid layer and an instability of the tear film. Therefore, the evaluation of lipid layer thickness (LLT) may be a useful tool in the diagnosis and classification of MGD.

Recently, the LipiView interferometer (TearScience Inc., Morrisville, NC, USA) was introduced to quantify LLT directly and non-invasively [6]. Several studies have evaluated the correlation between LLT and MG function in patients with MGD by using LipiView interferometer, and suggested that an LLT of ≤60 nm indicates the presence of MGD, while an LLT cut-off value of 75 nm has a higher diagnostic sensitivity [7, 8]. However, it is worth noting that age as an independent factor has been reported to influence the characteristics of the tear film lipid layer and tear film dynamics, and positively associated with LLT values in patients with dry eye syndrome [9, 10]. To date, there is a lack of data concerning these correlations between LLT and MG morphology and function in patients at different ages. Therefore, the purpose of this study was to investigate the relationship between the quantitative measurements of LLT by LipiView interferometer and other subjective and objective measurements in patients with obstructive MGD at different ages.

## Methods

### Subjects

In this cross-sectional study, subjects with obstructive MGD were consecutively recruited from the outpatient department of the Eye Hospital of Wenzhou Medical University, Hangzhou, China, between December 2016 and September 2017. Ethics approval was obtained from the Research Review Board at Wenzhou Medical University. The present study was conducted in accordance with the tenets of the Declaration of Helsinki and registered at ClinicalTrials.gov (NCT02481167). Written informed consent was provided by all patients before the examination.

The criteria for obstructive MGD diagnosis in accordance with previously reported [11]: 1) presence of ocular symptoms for at least 3 months; 2) changes in eyelid morphology including inflammation and swelling of the eyelid margin, telangiectasia, terminal duct obstruction, clogged meibomian gland orifices and keratinization or displacement of the mucocutaneous junction; and 3) poor quality and quantity of meibum expression. Patients aged less than 20 years and those had histories of ocular trauma or surgery (include the use of a punctal plug) within 3 months before the examinations, or had any disease affecting the ocular surface (i.e., ocular infection, allergy and systemic autoimmune disease) were excluded. Patients who wore contact lenses and used topical ocular medications other than artificial tears were also excluded. Patients were categorized into three subcategories according to their ages: young group (aged 20–39 years), middle-aged group (aged 40–59 years), and elderly group (aged ≥60 years).

### Outcome measures

Each patient was requested to complete a series of clinical examinations on both eyes, and data from the right eye were used for analysis. The clinical assessments were carried out in the following order: dry eye questionnaire, LLT, lower tear meniscus height (TMH), noninvasive breakup time (NIBUT), lid margin abnormality, corneal fluorescein staining (CFS), invasive tear breakup time (ITBUT), the Schirmer I test, expression and quality of meibum, and grading of MG dropout. An interval of 5 min was required between different tests. The room was maintained at 23–25 °C and 40–60% humidity during the examinations. All the above tests were performed by one of the authors (J.L.M).

### Ocular surface disease index

Subjective symptoms were assessed by the Ocular Surface Disease Index (OSDI) questionnaire [12]. The OSDI consists of 12 questions in the context of vision-related symptoms, ocular symptoms and environmental triggers, and each item was graded on a scale from 0 (none of the time) to 4 (all of the time). The scores on the OSDI questionnaire ranged from 0 to 100.

### Lipid layer thickness

**Fig. 1:**
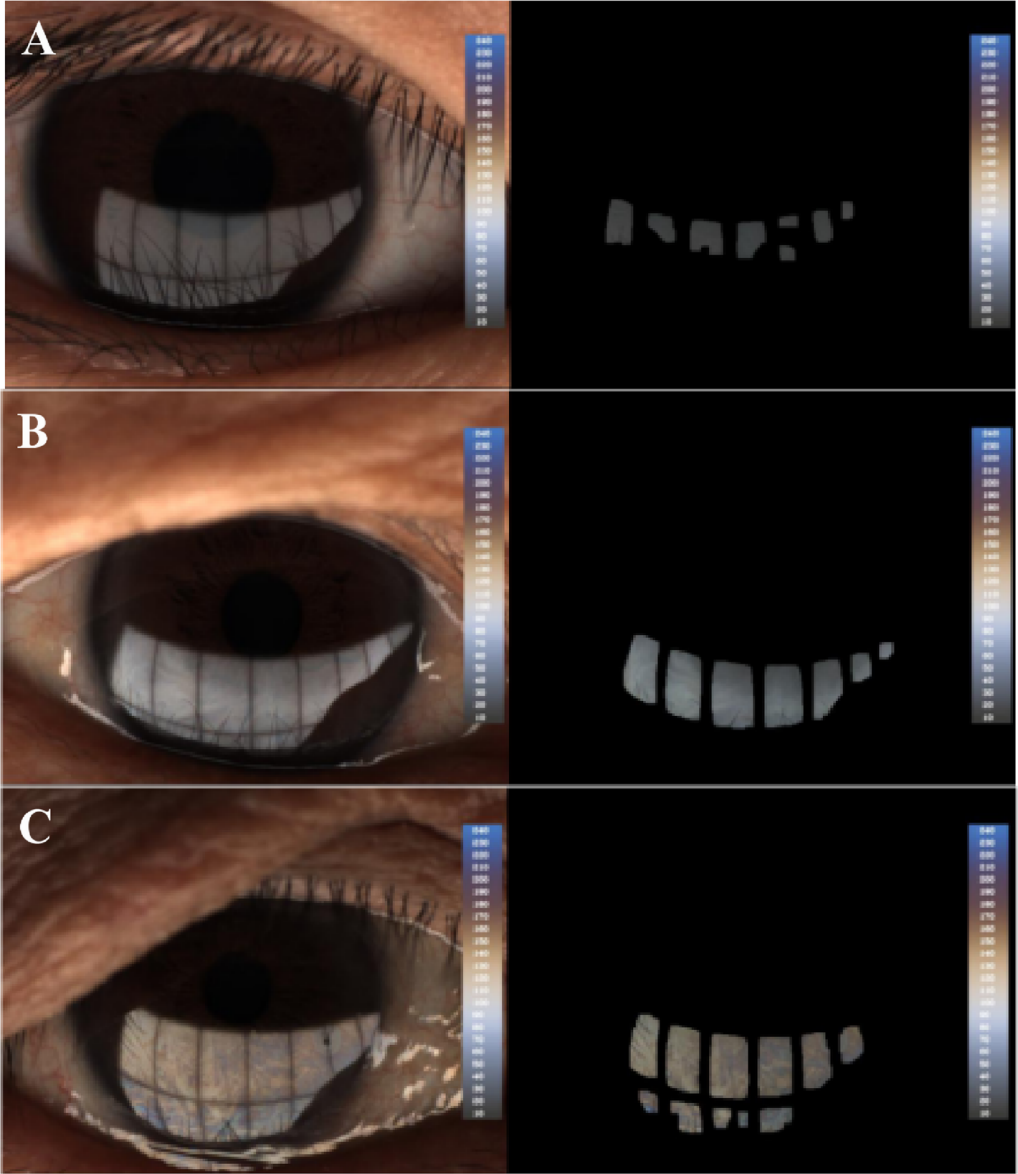
The analyzed image of lipid layer thickness (LLT) by the LipiView interferometer. The values are given in ICUs wherein 1 ICU approximately reflects 1 nm of the LLT. The average LLT of patient **a** was 32 nm, the average LLT of patient **b** was 72 nm and the average LLT of patient **c** was > 100 nm

LLT was quantified by using Lipiview Interferometer (TearScience Inc., Morrisville, NC) as previously described [9]. A 20-s video was captured to document the interference pattern of the tear film for each eye. The LLT deriving from the image is calculated as interferometric color units (ICU), where 1 ICU reflects approximately 1 nm (Fig. 1). The maximum LLT value is limited to be 100 nm.

### Tear meniscus height

The TMH was evaluated using the corneal topographer Keratograph 5 M (Oculus GmbH, Wetzlar, Germany) in a darkened room. Patients were asked to focus on the fixation target and a placido disc comprising of 22 mire rings was projected onto the corneal surface. The lower tear film meniscus images were captured 5 s after blinking, and the values of TMH were measured using an integrated ruler. All measurements were repeated three times and the average was recorded.

### Invasive tear breakup time

To evaluate the ITBUT, a fluorescein strip was quickly placed into the inferior conjunctiva. The time interval between the last complete blink and the appearance of first break spot on the corneal surface was recorded. The average of three consecutive ITBUTs was calculated.

### Corneal fluorescein staining

Three minutes after fluorescein instillation, CFS was evaluated using the slit-lamp microscope with a cobalt blue illumination. The cornea was divided into four quadrants (supertemporal, inferotemporal, supernasal, and inferonasal). The punctate epithelial erosions on corneal surface were scored on a scale of 0–3 in each quadrant: 0, no staining in the cornea; 1, < 5 punctuate staining; 2, > 5 punctuate staining but < 10; and 3, > 10 punctuate staining. A sum of the CFS scores ranged from 0 to 12.

### Schirmer I test

The Schirmer I test was performed on both eyes simultaneously without anesthesia. Schirmer strips were placed in the lower outer fornix and patients were instructed to close their eyes for 5 min. The strips were removed and the lengths of wetting were recorded.

### Eyelid margin abnormality

Eyelid margin abnormalities were scored as 0 (absent) or 1 (present) for the following four parameters: irregular lid margin, vascular engorgement, plugging of meibomian gland orifices, anterior or posterior displacement of the mucocutaneous junction [9]. The total scores were ranged from 0 to 4.

### Meibomian gland expressibility and secretion quality

The quantity and quality of MG secretion were assessed using the Meibomian Gland Evaluator (TearScience Inc.). A consistent pressure (about 1.25 g/mm^2^) was applied to five MGs in nasal, central, and temporal regions of the lower eyelid. Meibum secreted from each gland was scored from 0 to 3: 0, no secretion; 1, inspissated, like toothpaste; 2, cloudy fluid; 3, clear fluid. The total scores of 15 meibomian glands (range, 0–45) were summed [13].

### Meibomian gland loss

Meibography was performed at the lower lid of each eye using the Keratograph 5 M and analyzed using the polygon selection tool of ImageJ software (Version 1.50b, National Institutes of Health, USA) as described in detail previously [14]. The ratio of MG dropout area to the total area was recorded.

#### Statistical analysis

Statistical analyses were performed using SPSS for Windows (version 20.0; SPSS Inc., Chicago, IL, USA). The normal distribution of the data was confirmed first using the Kolmogorov-Smirnov test. The continuous and categorical data were described as the median with interquartile range (IQR) and frequency, respectively. Chi-square test was used to compare categorical variables, and the Kruskal-Wallis test with Dunn’s post hoc test and Bonferroni correction was used to compare the numeric variables. Spearman’s rank correlation was used to estimate the correlations between average LLT, MG dropout, and other clinical parameters because the most parameters were not normally distributed. Univariate and multivariate linear regression analyses were performed to assess the relationship between the LLT and age. *P* values less than 0.05 were accepted as significant.

## Results

A total of 209 eyes of 209 patients (132 females and 77 males; mean age, 49.68 ± 18.33 years) were included in this study. The demographics and parameters of all enrolled patients are summarized in Table 1. When analyzing demographics by age groups, we observed that the groups differed in age, OSDI score, LLT, eyelid margin abnormality, MGE score, Schirmer’s I test as well as central TMH (all *p* values ≤0.001; Kruskal-Wallis test).

**Table 1:** Demographics and clinical characteristics of the study subjects

Variables	All group (***n*** = 209)	Young group (***n*** = 77)	Middle-aged group (***n*** = 52)	Older group (***n*** = 80)	***P***
Age (y)	51 (33)	29 (8)	51 (7.75)	66 (13)	**< 0.001**
Sex (male/female)	77/132	28/49	18/34	31/49	0.885
OSDI score	23 (27)	31 (20.5)	29.5 (24.75)	14 (18.75)	**< 0.001**
LLT (nm)	57 (36.5)	51 (23.5)	59.5 (46.5)	62 (42.5)	**0.026**
Eyelid margin abnormality	2 (1)	2 (1)	2 (1)	2 (0)	**0.001**
MGE score	8 (10)	11 (9)	6 (11.25)	7.5 (8)	**0.001**
Schirmer’s I test (mm)	7 (12.5)	15 (19)	5 (7.75)	6 (6.75)	**< 0.001**
ITBUT (sec)	3 (2)	2.7 (2)	2.3 (1.23)	3 (2)	0.263
CFS score	1 (2)	1 (1)	1 (1)	1 (2)	0.745
NITBUT (sec)	4.4 (3.06)	4.7 (2.56)	4.2 (2.97)	4.0 (3.68)	0.712
Central TMH (mm)	0.18 (0.1)	0.19 (0.07)	0.19 (0.10)	0.18 (0.08)	**0.038**
MG dropout (%)	32 (12)	32 (11)	32 (13)	30 (13)	0.972

*OSDI* Ocular surface disease index; *LLT* Lipid layer thickness; *MGE* Meibomian gland evaluator, *ITBUT* Invasive tear break-up time; *CFS* Corneal fluorescein staining; *NITBUT* Non-invasive tear break-up time; *TMH* Tear meniscus height

All continuous data are expressed as medians (interquartile range, IQR)

Significant data are in bold faceik

**Fig. 2:**
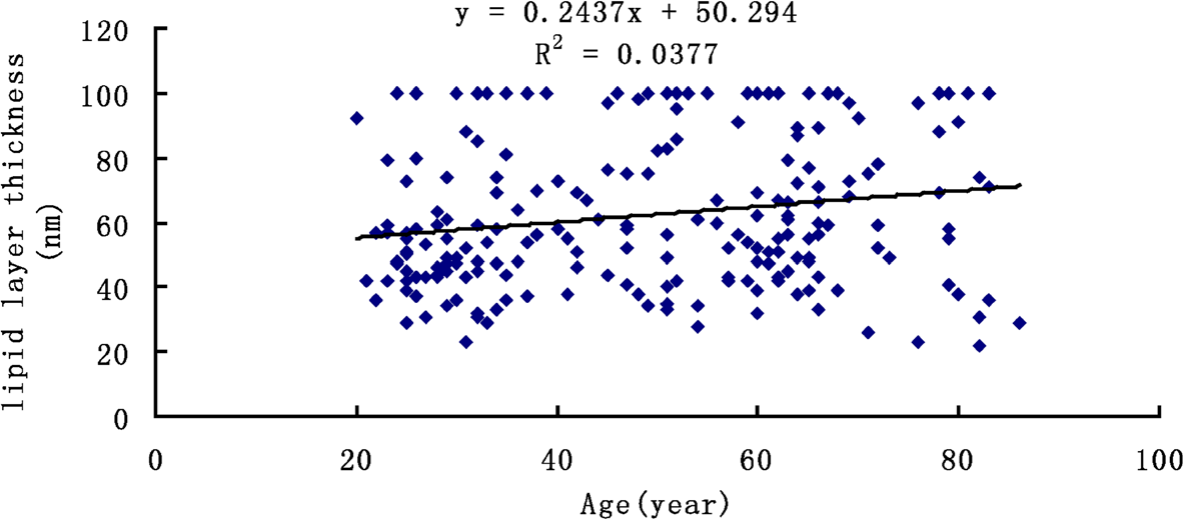
Association between the age (x-axis) and lipid layer thickness (y-axis), indicating a significant positive Spearman correlation (y = 0.244 + 50.294, *r* = 0.190, *P* = 0.006)

The median average LLT values were 57 nm (IQR, 36.5 nm), 51 nm (IQR, 23.5 nm), 59.5 nm (IQR, 46.5 nm) and 62 nm (IQR, 42.5) in the all patients group, young group, middle-aged group and older group, respectively. A linear regression of age and LLT revealed a positively weak correlation between them (y = 0.24x + 50.29, *r* = 0.190, *P* = 0.006; Fig. 2). After adjustment for confounding factors (MG dropout and sex) in multivariate regression, the correlation was significantly remained (β = 5.539, *P* = 0.001; Table 2).

**Table 2:** Coefficients for multivariate regression model

Variables	Unstandardized Coefficients		Standardized Cofficients		
	B	Std. Error	Beta	t	Sig.
Constant	79.679	7.499		10.625	**< 0.001**
Age group	5.3599	1.572	−0.203	3.408	**0.001**
Sex	5.651	2.820	0.119	2.004	**0.046**
MG dropout	−116.359	14.558	−0.475	−7.993	**< 0.001**

Dependent Variable: Lipid layer thickness

Significant data are in bold face

The correlation analyses between the LLT and other objective measurements in different aged group are summarized in Table 3. Spearman correlation analysis showed that the LLT was remarkably negatively correlated with MG dropout in all patients (*r* = − 0.519, *P* < 0.001), young group (*r* = − 0.536, *P* < 0.001), middle-aged group (*r* = − 0.576, *P* < 0.001) and older aged group (*r* = − 0.501, *P* < 0.001). In contrast, LLT was positively correlated with MGE score in all patients (*r* = 0.202, *P* = 0.003), middle-aged group (*r* = 0.280, *P* = 0.044), older group (*r* = 0.452, *P* < 0.001), but not in young group (*r* = 0.007, *P* = 0.949).

**Table 3:** Correlations between average LLT and clinical parameters

Variables	All group (***n*** = 209)		Young group (***n*** = 77)		Middle-aged group (***n*** = 52)		Older group (***n*** = 80)	
	***r***	***P***	***r***	***P***	***r***	***P***	***r***	***P***
OSDI score	−0.035	0.614	−0.020	0.866	−0.015	0.917	0.026	0.820
Lid margin abnormality	0.018	0.792	0.076	0.513	−0.189	0.181	−0.014	0.901
MGE score	0.202	**0.003**	0.007	0.949	0.280	**0.044**	0.452	**< 0.001**
Schirmer’s I test (mm)	−0.067	0.338	0.026	0.824	0.186	0.187	−0.134	0.235
ITBUT (sec)	−0.007	0.919	− 0.097	0.403	−0.058	0.682	0.148	0.189
CFS score	−0.135	0.052	−0.064	0.581	−0.156	0.269	−0.200	0.075
NITBUT (sec)	0.020	0.778	−0.040	0.732	0.115	0.418	0.026	0.816
Central TMH (mm)	−0.067	0.336	−0.066	0.571	−0.018	0.898	−0.007	0.498
MG dropout (%)	−0.527	**< 0.001**	−0.536	**< 0.001**	− 0.576	**< 0.001**	−0.501	**< 0.001**

*OSDI* Ocular surface disease index; *LLT* Lipid layer thickness; *MGE* Meibomian gland evaluator, *ITBUT* Invasive tear break-up time; *CFS* Corneal fluorescein staining; *NITBUT* Non-invasive tear break-up time; *TMH* tear meniscus height

Significant data are in bold face

The correlation analyses between the MG dropout and other objective measurements excepting for the LLT in different aged group are summarized in Table 4. We found that MG dropout was negatively correlated with LLT in all MGE score in all patients (*r* = − 0.133, *P* = 0.045), but the correlations were not significant in any group. Moreover, the MG dropout did not differ among three groups (*P* = 0.972; Kruskal-Wallis test).

**Table 4:** Correlations between meibomian gland dropout and clinical parameters

Variables	All group		Young group (***n*** = 77)		Middle-aged group (***n*** = 52)		Older group (***n*** = 80)	
	***r***	***P***	***r***	***P***	***r***	***P***	***r***	***P***
OSDI score	0.077	0.270	0.196	0.088	0.122	0.388	0.021	0.2854
Lid margin abnormality	0.019	0.783	−0.060	0.606	0.229	0.102	−0.013	0.905
MGE score	−0.133	**0.045**	−0.014	0.903	−0.193	0.171	−0.192	0.088
Schirmer’s I test (mm)	0.027	0.694	0.046	0.692	−0.099	0.487	0.078	0.493
ITBUT (sec)	0.009	0.894	0.054	0.644	−0.043	0.765	0.015	0.894
CFS score	0.110	0.112	−0.028	0.810	0.251	0.072	0.135	0.233
NITBUT (sec)	0.035	0.614	0.059	0.610	−0.188	0.183	0.136	0.228
Central TMH (mm)	0.009	0.899	0.120	0.300	0.076	0.594	−0.092	0.415

*OSDI* Ocular surface disease index; *LLT* Lipid layer thickness; *MGE* Meibomian gland evaluator, *ITBUT* Invasive tear break-up time; *CFS* Corneal fluorescein staining; *NITBUT* Non-invasive tear break-up time; *TMH* Tear meniscus height

Significant data are in bold face

## Discussion

It has been reported that MGD-related dry eye is present in 20 to 69% of patients [3]. Any abnormity in the component of lipid layer or the secretory system of the MGs, as in obstructive MGD, will markedly increase water evaporation from the ocular surface. Therefore, the thickness of the lipid layer could be used as a representative parameter of the MG function, and LLT assessment was a useful tool for obstructive MGD diagnosis.

In current study, the median value of LLT was 57 nm, which was thinner than the results in previous studies conducted by Jung et al. [9] and Sang et al. [15], in which the median LLT values in eyes with obstructive MGD were 79 nm and 75 nm, respectively. On the contrary, our results were similar to the study reported by Eom et al. [7], who used the same instrument to measure the LLT in patients with obstructive MGD. Although LLT measured with the LipiView interferometer has been proved to be relatively constant [16], demographic factors such as age and sex may be the confounding factors that influence the ILL values [9, 10]. Jung et al. found that age as an independent factor associated with increased LLT in normal subjects as well as in patients with dry eye syndrome [9]. Consistent with their report, we observed that LLT positively correlates with age (β = 5.539, *P* = 0.001) in the patients with obstructive MGD after adjustment for MG dropout and sex. Therefore, we categorized all patients into three subcategories (young, middle-aged and older) in further analyses.

A positive correlation between the LLT measurement and expressible MGs has been reported in several studies [7, 8, 15, 17], indicating that the LLT assessments well reflect the quantitative changes of the meibum expression. Our data demonstrated that the LLT correlated well with MGE score in middle-aged and older groups, but not in young group. Considering that the MG secretion is more vigorous in young patients, when some glands are blocked, the secretory capacity of the residual meibomian glands may be compensatorily increased to maintain the tear film LLT within the normal range.

In the present study, LLT was significantly negatively correlated with MG dropout regardless of patients’ age. This finding is similar to the results of previous studies which demonstrated a significant correlation between LLT and both upper and lower MG dropout grade in patients with MGD as well as normal controls [7, 17]. Moreover, the negative correlation between MG dropout and MGE score (*r* = − 0.133, *P* = 0.045), strongly indicates MG dropout is associated with an impaired MG function. Interestingly, correlations between MG dropout and MGE were stronger in the older group (*r* = − 0.192, *P* = 0.088) than that in the young and middle-aged groups (*r* = − 0.014, *P* = 0.903; *r* = − 0.193, *P* = 0.171). These results highlight that the aging process is accompanied with functional and morphological MG alterations [18, 19]. Based on the correlation among LLT, MGE and MG dropout in the middle-aged and elderly groups, we believe that LLT combined with MG dropout can be used as an objective indicator for MGD diagnosis, and partially avoid to perform the invasive examination of MGE. However, additional investigation is needed to evaluate the fundamental mechanisms and causes of MG dropout.

In this study, we also found that LLT was not correlated with other parameters. Although an increase in tear fluid production compensating for the deficiency of the oily layer resulting from MG loss has been reported by a multicenter cross-sectional study [20], the correlation between LLT and tear production remains controversial and needs further investigation.

There were some limitations in this study. First, no healthy controls were included in this study, which prevent us from assessing whether there is a significant difference in LLT between healthy and disease states. Second, because the expression of Meibomian glands may be a potential confounding factor [16], further studies are needed to determine the correlation between the LLT and different categories of MGD.

## Conclusion

In summary, our data show that LLT was positively correlated with MGE, but negatively correlated with MG dropout in patients with MGD. LLT measurement is a useful tool for the assessment of MGD, and age as a strong influential factor should be taken to account when interpreting the meaning of the LLT value.

## Data Availability

The datasets used and/or analyzed during the current study are available from the corresponding author upon reasonable request.

## Abbreviations

MGD: Meibomian gland dysfunction
LLT: Lipid layer thickness
OSDI: Ocular surface disease index
TMH: Tear meniscus height
NIBUT: Noninvasive breakup time
CFS: Corneal fluorescein staining
ITBUT: Invasive tear breakup time
ICU: Interferometric color units

## References

[1] Korb DR, Henriquez AS (1980). Meibomian gland dysfunction and contact lens intolerance. J Am Optom Assoc.

[2] Nichols KK, Foulks GN, Bron AJ, Glasgow BJ, Dogru M, Tsubota K (2011). The international workshop on meibomian gland dysfunction: executive summary. Invest Ophthalmol Vis Sci.

[3] Schaumberg DA, Nichols JJ, Papas EB, Tong L, Uchino M, Nichols KK (2011). The international workshop on meibomian gland dysfunction: report of the subcommittee on the epidemiology of, and associated risk factors for, MGD. Invest Ophthalmol Vis Sci.

[4] Giannaccare G, Vigo L, Pellegrini M, Sebastiani S, Carones F (2018). Ocular surface workup with automated noninvasive measurements for the diagnosis of Meibomian gland dysfunction. Cornea.

[5] Millar TJ, Schuett BS (2015). The real reason for having a meibomian lipid layer covering the outer surface of the tear film - a review. Exp Eye Res.

[6] Blackie CA, Solomon JD, Scaffidi RC, Greiner JV, Lemp MA, Korb DR (2009). The relationship between dry eye symptoms and lipid layer thickness. Cornea.

[7] Eom Y, Lee JS, Kang SY, Kim HM, Song JS (2013). Correlation between quantitative measurements of tear film lipid layer thickness and meibomian gland loss in patients with obstructive meibomian gland dysfunction and normal controls. Am J Ophthalmol.

[8] Finis D, Pischel N, Schrader S, Geerling G (2013). Evaluation of lipid layer thickness measurement of the tear film as a diagnostic tool for Meibomian gland dysfunction. Cornea.

[9] Jung JW, Park SY, Kim JS, Kim EK, Seo KY, Kim TI (2016). Analysis of factors associated with the tear film lipid layer thickness in Normal eyes and patients with dry eye syndrome. Invest Ophthalmol Vis Sci.

[10] Maissa C, Guillon M (2010). Tear film dynamics and lipid layer characteristics--effect of age and gender. Cont Lens Anterior Eye.

[11] Asbell PA, Stapleton FJ, Wickstrom K, Akpek EK, Aragona P, Dana R (2011). The international workshop on meibomian gland dysfunction: report of the clinical trials subcommittee. Invest Ophthalmol Vis Sci.

[12] Schiffman RM, Christianson MD, Jacobsen G, Hirsch JD, Reis BL (2000). Reliability and validity of the ocular surface disease index. Arch Ophthalmol.

[13] Zang S, Cui Y, Cui Y, Fei W. Meibomian gland dropout in Sjogren's syndrome and non-Sjogren's dry eye patients. Eye (Lond). 2018:32:1681–87. 10.1038/s41433-018-0149-5 PMC6224582 29934634

[14] Zhao Y, Xie J, Li J, Fu Y, Lin X, Wang S (2016). Evaluation of monocular treatment for Meibomian gland dysfunction with an automated thermodynamic system in elderly Chinese patients: a contralateral eye study. J Ophthalmol.

[15] Sang X, Li Y, Yang L, Liu JH, Wang XR, Li CY (2018). Lipid layer thickness and tear meniscus height measurements for the differential diagnosis of evaporative dry eye subtypes. Int J Ophthalmol.

[16] Finis D, Pischel N, Borrelli M, Schrader S, Geerling G (2014). Factors influencing the measurement of tear film lipid layer thickness with interferometry. Klin Monatsbl Augenheilkd.

[17] Ji YW, Lee J, Lee H, Seo KY, Kim EK, Kim TI (2017). Automated measurement of tear film dynamics and lipid layer thickness for assessment of non-Sjogren dry eye syndrome with Meibomian gland dysfunction. Cornea.

[18] Pult H (2018). Relationships between Meibomian gland loss and age, sex, and dry eye. Eye Contact Lens.

[19] Machalinska A, Zakrzewska A, Safranow K, Wiszniewska B, Machalinski B (2016). Risk factors and symptoms of Meibomian gland loss in a healthy population. J Ophthalmol.

[20] Arita R, Morishige N, Koh S, Shirakawa R, Kawashima M, Sakimoto T (2015). Increased tear fluid production as a compensatory response to Meibomian gland loss: a multicenter cross-sectional study. Ophthalmology.

